# DENTINE MATRIX METALLOPROTEINASES AS POTENTIAL MEDIATORS OF DENTINE REGENERATION

**DOI:** 10.22203/eCM.v042a24

**Published:** 2021-11-24

**Authors:** E. Guirado, A. George

**Affiliations:** Department of Oral Biology, University of Illinois at Chicago College of Dentistry, Chicago, USA

**Keywords:** Dentine, enamel, periodontal ligament, tooth, dental regenerative repair, matrix metalloproteinases, dental physiology, signalling molecules/growth factors

## Abstract

Matrix metalloproteinases (MMPs) have been implicated not only in the regulation of developmental processes but also in the release of biologically active molecules and in the modulation of repair during tertiary dentine formation. Although efforts to preserve dentine have focused on inhibiting the activity of these proteases, their function is much more complex and necessary for dentine repair than expected. The present review explores the role of MMPs as bioactive components of the dentine matrix involved in dentine formation, repair and regeneration. Special consideration is given to the mechanical properties of dentine, including those of reactionary and reparative dentine, and the known roles of MMPs in their formation. MMPs are critical components of the dentine matrix and should be considered as important candidates in dentine regeneration.

## Tissue structure and function in regeneration

The tightly correlated structure-function relationship in physiological dentine requires that regeneration of the tissue approximate primary dentine as closely as possible. In other words, dentine regeneration necessitates the preservation of the mechanical properties of the tissue rendered by its ultrastructure. Dentine is a mineralised, but vital, tissue housing the cellular processes of odontoblasts within dentineal tubules and responsible for dissipating the stresses of mastication (Goldberg et al., 2011). These forces must be transferred from a stiff (enamel, 96 % mineral, by weight) to a more elastic (dentine, 70 % mineral, by weight) material. Dentine matrix is composed of collagenous (86 % type I, as well as types III, V and VI) and non-collagenous proteins.

Dentine is capable of limited repair following pulpal insult from carious lesions, cavity preparations, erosion and restorative dental materials. Dentine repair through tertiary dentine deposition results in inward growth of the circumpulpal dentine layer constricting the pulp chamber and root canals. Tertiary dentine does not preserve the precise tubular system of physiological dentine. Minimal trauma that spares the underlying odontoblast layer or Hoehl’s cells results in reactionary dentine formation, in the form of tubular orthodentine or atubular, bone-like osteodentine (Goldberg et al., 2015). Reactionary dentine has reduced hardness and lower elasticity, despite being heavily mineralised (Charadram et al., 2013; Senawongse et al., 2006). More severe insults that result in cellular death require progenitor cells to repair the defect by depositing reparative dentine in the form of a dentineal bridge. It is unclear whether the amount of tertiary dentine present in a tooth affects its function.

Dentine hardness is directly proportional to the mineral density of the tissue and has a linear correlation with lack of elasticity. The mineral density varies across the tissue, with the most mineral dense, hardest and least elastic dentine near the dentineal tubule edge, called peritubular dentine (MacDougall et al., 1992). Between tubules lies the more elastic intertubular dentine. Hardness is lowest near the DEJ, peaks in circumpulpal dentine and again decreases towards the pulp (Wang and Weiner, 1997). Dentineal tubules become more abundant, closer together and larger as they approach the odontoblastic cell body (Pashley et al., 1985). Changes in dentineal tubule density correlate with changes in the ratios of intertubular and peritubular dentine, thus affecting the hardness and the mechanical properties of the tissue.

To the authors’ knowledge, no studies have shown whether MMP activity affects tubular density and the physical and mechanical properties of tertiary dentine. Due to the importance of these factors to dentine function, future regenerative models should consider using them as indicators of success.

### What’s dentine regeneration and what does it entail?

Dentine repair and regeneration invariably depend on pulp vitality. Regenerative endodontics has used this concept in the treatment of immature teeth with open apices. The dental pulp is a loose connective tissue composing the core of the tooth structure. It consists of cellular (fibroblasts, odontoblasts, immune cells, neurovascular networks) and extracellular (collagen, fibronectin, glycoproteins) components that exist in close relationship with dentine (Goldberg et al., 2011). Vital pulp therapies promote the regeneration of vascularised, innervated dental pulp able to support odontoblast differentiation and dentine neogenesis for the completion of root formation (Huang, 2011). Dentine regeneration in such a form fails to address the loss of clinical crown due to caries. Studies do not clarify the amount of dentine that can potentially be replaced or whether these equal the amount lost due to disease. Furthermore, due to the acellular nature of the enamel, regeneration of this tissue poses even more challenges than dentine regeneration (Pandya and Diekwisch, 2019).

Tissue regeneration demands, at minimum, the potential for replacement of cellular and matrix components through proliferation (Krafts, 2010). Dentine regeneration will require replacement of carious dentine matrix by newly formed proliferating odontoblasts and differentiating dental pulp stem cells. To this end, scaffolds along with biological cues pose the most promise allowing for subsequent cellular infiltration, matrix deposition and mineralisation.

Alginates, chitosan, hyaluronic acid, collagen, gelatine, fibrin, silk and hydrogel scaffolds have been effective in supporting dental pulp cell growth and differentiation (Inuyama et al., 2010; Panseri et al., 2016; Prescott et al., 2008; Vagropoulou et al., 2021; Yang et al., 2015). Although these scaffolds promise to promote pulpal regeneration, no studies have shown appreciable levels of mineralisation to replace the amount of dentine lost due to caries. Moreover, the time required to regenerate the bulk tissue destroyed by caries would be extensive and clinically unacceptable. The tooth may need to be out of occlusion to prevent excessive forces from disrupting the regeneration process. Also, it would probably require a full-coverage restoration for better long-term prognosis.

## Dentine MMPs

MMPs are a family of 28 modular endopeptidases involved in extracellular matrix remodelling and regulation of extracellular signalling networks guiding inflammation, bone development and angiogenesis, among others (Löffek et al., 2011). They are produced by the cellular components of soft and hard tissues, including epithelial cells, endothelial cells, fibroblasts, osteoclasts, osteoblasts, hypertrophic chondrocytes, chondroclasts, inflammatory cells and odontoblasts (Goldberg et al., 2003; Ortega et al., 2003; Sulkala et al., 2002). Most MMPs contain a propeptide domain, responsible for preserving the latent conformation of the enzyme; a zinc-binding catalytic domain, responsible for their proteolytic function; a haemopexin-like domain, responsible for proteinprotein interactions. These zymogens are activated by a variety of mechanisms, both proteolytic and nonproteolytic, that make them suitable for their different functions (Van Wart and Birkedal-Hansen, 1990). They can be classified by their substrates, which are largely determined by specificity-determining positions on their catalytic domain (Ratnikov et al., 2014). The MMPs found in dentine include MMP-2, -3, -7, -8, -9, -13, -14, -20, -23 and -25 (Eckhard et al., 2015; Goldberg et al., 2003; Hall et al., 1999; Loreto et al., 2014; Mazzoni et al., 2007, 2018; Sulkala et al., 2002, 2007; Xu et al., 2016) (Table 1).

The dynamics of MMP activation and inhibition remain ill-defined, albeit one of the most important features of tissue remodelling in response to disease. Historically, MMP proteolytic activity has been associated with tissue destruction. MMP-8 has been identified as one of the main collagenases in human dentine involved in the caries process, while increases in MMP-14 activity have also been associated with a response to caries, although its exact role remains unclear (Charadram et al., 2012; Sulkala et al., 2007; Tjäderhane et al., 2015). These endogenous MMPs may be released from the extracellular matrix and/or activated by the caries process and are likely to sustain the disease through their enzymatic activity. Moreover, their loss of function has been associated with decreased caries risk, as in the case of MMP-13 (Loreto et al., 2014; Tannure et al., 2012).

Efforts to slow or prevent disease have focused on inhibiting MMP activity. Failure of resin-based restorations due to hybrid layer degradation has been attributed to the enzymatic degradation of collagen fibrils by MMPs. To date, the use of exogenous MMP inhibitors, such as tetracycline antibiotics and chlorhexidine, have improved the clinical outcomes of resin-bonded restorations by preserving the hybrid layer and bond strength (Breschi et al., 2018; Gendron et al., 1999; Hanemaaijer et al., 2001).

Endogenous TIMP-1 to -4 have been identified in human dentine. TIMP-2 expression increases during the caries process, although this increase is concurrent with enhanced MMP expression (Charadram et al., 2012; Goldberg et al., 2003). The significance of their co-expression has yet to be determined, although MMP/TIMP ratios and substrate-inhibitor specificity may explain and control MMP activity in tissues. TIMP signalling independent of its MMP-inhibitory action has also been proposed. TIMP-1 and -2 expression are important components of scaffolds used in regenerative endodontic procedures (Koh et al., 2007; Suresh et al., 2018). Furthermore, growth factors that contribute to pulp cell proliferation are known to stimulate TIMP expression (Chang et al., 2017). In such cases, MMP co-expression may be explained by MMP counter-regulatory action on TIMP signalling. Ultimately, regeneration requires a balance in matrix turnover. Leveraging MMP activity would require a better understanding of MMP-TIMP protein interactions and biological functions.

MMPs play a crucial role during tooth development. MMP-2 and -9 are the earliest MMPs to be expressed and may be involved in the degradation of the basement membrane, marking the onset of the terminal differentiation of ameloblasts and odontoblasts (Heikinheimo and Salo, 1995). While important at early stages, MMP-2 (and MMP-20) loss of function results in higher levels and broader distribution of non-collagenous proteins known to promote dentine mineralisation (Bourd-Boittin et al., 2005). Still at later stages of development, the loss of function of other MMPs, such as MMP-14, results in root dentine defects (Xu et al., 2016). Evidence indicates that MMPs are not redundant proteins with interchangeable functions. Their specificities of action can mediate tissue development and their dysregulation carries significant implications for tissue integrity.

Dentine regeneration and tertiary dentine formation depend on the appropriate signalling molecules’ – potential for cell differentiation, migration and proliferation – and appropriate remodelling of newly deposited dentine matrix. MMPs are crucial for these processes. Thus, it might not be feasible to completely abolish their proteolytic activity to achieve regeneration. In fact, certain MMPs, although present during disease, may play a protective role by facilitating the repair process. MMP-3 exhibits optimal proteolytic activity and calcium affinity in a pH-dependent manner (range pH 5.3-5.5). Thus, its optimal proteolytic activity lies at the critical pH of demineralisation of enamel and dentine, such as those found in carious environments (Abou Neel et al., 2016; Wilhelm et al., 1993). MMP-3, however, has been associated with angiogenesis and reparative dentine deposition (Zheng et al., 2009).

### Leveraging MMPs for dentine regeneration

MMPs have been implicated not only in the regulation of developmental processes, but also in the release of biologically active molecules and modulation of repair during tertiary dentine formation (Chaussain-Miller et al., 2006; Sternlicht and Werb, 2001). MMPs clear the way for incoming progenitor cells as well as activate growth factors responsible for angiogenesis, immune regulation and cellular differentiation. MMP activity in response to disease may arise from exogenous (bacterial products) and endogenous (immune cells) sources as well as from dentine matrix reservoirs. Bacterial products from carious lesions may also lead to signalling cascades that activate MMP secretion by odontoblasts. Odontoblasts adjacent to reactionary dentine express high levels of MMP-2, which has been associated with increased proteolytic activity in this zone (Charadram et al., 2012). This activity could be responsible for the maturation of collagen fibres and the initiation of mineral formation in the newly formed dentine (Jontell and Linde, 1983).

MMPs contribute to debris clearance and new tissue formation. Animal models of tissue regeneration support the importance of these enzymes in the regenerative process. MMP upregulation is one of the earliest steps in newt limb regeneration after amputation. MMP activity is essential and its inhibition stunts limb regeneration (Vinarsky et al., 2005). MMP-9, -3 and -13 were the most highly expressed in this particular model (Miyazaki et al., 1996). Similarly, pulp injury results in an inflammatory response characterised by the invasion of polymorphonuclear cells and the release of proteases, such as MMP-9 (Gusman et al., 2002; Mente et al., 2016). These MMPs contribute to the degradation of exposed carious dentine, angiogenesis and cell migration, thus activating pathways that lead to tertiary dentine deposition (Charadram et al., 2012). MMP-9 is currently being used in endodontics as a diagnostic and prognostic measure of pulpal inflammation to help guide clinical decisions (Mente et al., 2016; Sharma et al., 2020; Zehnder et al., 2011).

*In vivo*, MMP-3 treatment results in reversal of mild irreversible pulpitis partly due to its anti-inflammatory properties. These properties include the inhibition of IL-6 expression and a decrease in macrophage and antigen-presenting cell infiltration (Eba et al., 2012). Furthermore, MMP-3 is able to stimulate CTGF production, independent of its proteolytic activity, thus enhancing the migration of dental pulp cells (Muromachi et al., 2012; VanHook, 2008). MMP-3 has also been localised to endothelial cells and stimulates angiogenesis and reparative dentine deposition in pulp injury models, *in vivo* (Zheng et al., 2009). Increased MMP-3 activity is not found in irreversibly injured pulps further supporting a regenerative role for the protein (Gusman et al., 2002). Future models of dentine regeneration should especially consider MMP-3 as a mediator of regeneration.

Lining the periphery of the pulp chamber is a palisade layer of odontoblasts responsible for the formation of dentine. During dentine deposition, these cells secrete bioactive molecules that orchestrate the mineralisation of the tissue. Similarly, in response to dentine destruction resulting from attrition, carious exposures and chemical insults, these cells are stimulated to secrete new dentine. However, during regeneration and repair, the chemical signals and cellular dynamics responsible for primary dentine formation are absent. Thus, the tissue relies on bioactive molecules to stimulate the proliferation, migration and differentiation of cells responsible for dentine regeneration and repair (Abbass et al., 2020).

**Fig. 1.**
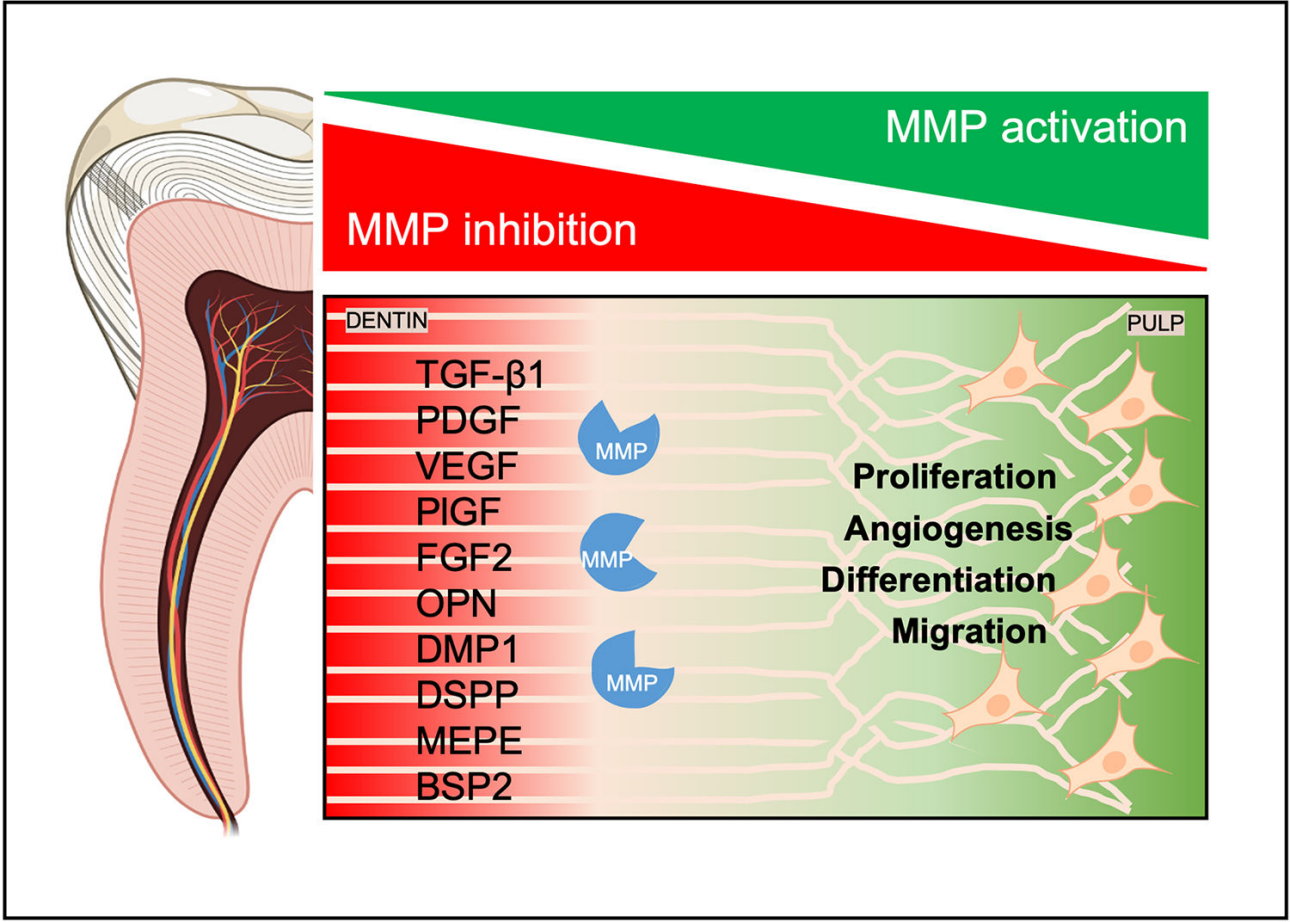
Dentine is a reservoir of bioactive molecules. MMPs are a family of proteinases responsible for dentine repair during environmental insults to the tooth, such as dental caries. MMPs orchestrate the activation of non-collagenous proteins and signalling molecules that, in turn, stimulate angiogenesis as well as proliferation, differentiation and migration of dental pulp cells during dentine repair.

Mature dentine is a reservoir of bioactive molecules once involved in its physiological deposition. As such, the tissue contains within it a defence mechanism for environmental insults. TGF-β1, PDGF-AB, VEGF, PlGF and FGF2 are found sequestered in dentine (Bègue-Kirn et al., 2004; Roberts-Clark and Smith, 2000; Zhao et al., 2000) (Fig. 1). Solubilisation of these growth factors promote angiogenesis, odontoblast differentiation and tertiary dentine deposition; ultimately resulting in dentineal bridges that are thicker, denser and structurally similar to physiological dentine (Galler et al., 2011; Liu et al., 2021). Furthermore, isolation of these factors from plasma can sustain tooth-bud cell viability and has resulted in the regeneration of teeth in porcine animal models (Yang et al., 2012). Dentine conditioning with EDTA is a promising strategy to growth factor release and stimulation of gene expression that potentiates odontoblast differentiation (Sadaghiani et al., 2016). MMPs provide another method for the release of these factors from dental tissues. The use of phosphoric acid etch-and-rinse adhesive systems has the potential to expose these proteases while maintaining their function (Tezvergil-Mutluay et al., 2013). Promotion of pulp healing has been associated with many of these proteases. Direct pulp capping agents consisting of MMP-digested dentine matrix components have shown similar regenerative properties *in vivo* (Okamoto et al., 2018). These studies identified MMP-1, -9, -13 and -20 as promoters of pulpal healing. Endogenous MMP activity has been leveraged in the delivery of growth factors from scaffolds in models of tissue regeneration. In a recent study, Huang et al. (2020) have engineered a scaffold containing an MMP-2 cleavage site and heparinbinding sites to bind growth factors. Such hydrogel scaffolds would be useful for the release of growth factors when activated by MMP2 *in vivo*.

Concentrated growth factors from venous blood, consisting of PDGF-BB, TGF-β1, VEGF and others, have been shown to inhibit proinflammatory cytokine release by dental pulp cells and result in the regeneration of dentine-pulp complex, *in vivo* (Xu et al., 2019). Immunomodulation of the pulpal inflammatory response to injury has also been achieved using melatonin-induced dental pulp TGF-β secretion (García-Bernal et al., 2020). Overexpression of PDGF-BB promotes regeneration through recruitment of dental pulp stem cells, enhancing their proliferation and odontogenic differentiation as well as enhancing pulp angiogenesis (Zhang et al., 2017). Similarly, bioactive pulp-capping agents containing BMPs, such as BMP-2 and -4, as well as FGF2 induce cell differentiation and tertiary dentine deposition (Ishimatsu et al., 2009; Nakashima, 1994).

Cells, signalling molecules and scaffolds form the tissue engineering triad. Successful scaffolds must persist in tissues for long enough to allow for cellular colonisation, after which they must undergo enzyme-mediated degradation (Murphy et al., 2013). MMPs have been leveraged in the timely removal of hydrogel scaffold systems, thereby sustaining cell colonisation and long-term proliferation, qualities essential for neovascularisation and angiogenesis (Lutolf et al., 2003; Turturro et al., 2013). Native cells within the regenerating tissues are crucial for maintaining the scaffolds. Secretion of anti-inflammatory cytokines, such as IL-10, induces TIMP expression and prevents scaffold degradation by MMPs (Ye et al., 2011). Conversely, inflammatory cells within the regenerating tissues are also known to secrete MMPs responsible for remodelling these scaffolds and newly deposited extracellular matrices (Hong et al., 2017).

As stated above, MMPs are instrumental in the formation of tertiary dentine. These enzymes function in the maturation of the dentine collagen matrix, enhancing the bioavailability of signalling molecules, and the cellular events that result in dentine repair. Activation of signalling molecules is particularly important in the mineralisation process. In addition to maturation of the collagenous components of the dentine matrix, MMPs are involved in the maturation and function of a group of non-collagenous proteins called SIBLINGs. Proteins in this family include OPN, DMP1, DSPP, MEPE and BSP2.

SIBLINGs bind specifically to MMPs, activating both latent MMPs and TIMP-inhibited MMPs (Fedarko et al., 2004). The known partners include OPN/MMP-3, DMP1/MMP-9 and BSP/MMP-2. MMP- 2 can also cleave DMP1 to release biologically active peptides and MMP-9 can cleave DSPP into DSP and DPP (Chaussain et al., 2009; Yuan et al., 2017). DSPP and MEPE have no MMP partner known to date (Ogbureke and Fisher, 2004). SIBLINGs represent most of the phosphorylated extracellular matrix proteins in dentine and have been implicated in mineralisation and odontoblast differentiation (Almushayt et al., 2006; Eapen and George, 2015). The relationship between these two protein families may represent an opportunity for dentine repair.

## Conclusion

MMPs are a family of proteinases responsible for matrix maturation and remodelling as well as modulation of non-collagenous proteins and signalling molecules that result in dentine repair. These proteinases play a developmental role during disease and repair, having functions as diverse as the protein family itself. The existing literature shows that MMPs do not all have similar properties. Some can have a more destructive while others a more regenerative function. To date, dentine regeneration has been approached in either of two ways: (1) by stimulating dentine deposition by stem cells; (2) by generating scaffolds to facilitate the deposition of mineralised tissue at the site of the dentine defect. The spatiotemporal regulation of MMP expression, their multifunctional properties and their ability to autoregulate make these multifaceted proteins ideal candidates for stimulation of dentine regeneration. Future research should focus on leveraging the properties of these enzymes in the promotion of dentine regeneration.

## Figures and Tables

**Table 1.** Roles of dentine MMPs. Endogenous MMPs have been associated with caries susceptibility and dentine repair potential. Evidence indicates that MMPs are not redundant proteins with interchangeable functions.

Dentine MMP	Functions	References
MMP-2	Associated with developmental processes and caries; known to bind bone sialoprotein; cleaves dentine matrix protein 1	Charadram et al., 2012 Goldberg et al., 2003 Mazzoni et al., 2007
MMP-3	Associated with angiogenesis and reparative dentine deposition; known to bind osteopontin	Eba et al., 2012 Hall et al., 1999 Muromachi et al., 2012 VanHook, 2008 Zheng et al., 2009
MMP-7	Role remains unclear	Mazzoni et al., 2018
MMP-8	Associated with caries	Sulkala et al., 2007
MMP-9	Associated with developmental processes and caries; known to bind dentine maxtrix protein 1; cleaves dentine sialophosphoprotein	Charadram et al., 2012 Goldberg et al., 2003 Mazzoni et al., 2007 Okamoto et al., 2018
MMP-13	Associated with increased caries risk	Loreto et al., 2014 Okamoto et al., 2018 Tannure et al., 2012
MMP-14	Role remains unclear	Xu et al., 2016
MMP-20	Role remains unclear	Okamoto et al., 2018 Sulkala et al., 2002
MMP-23	Role remains unclear	Eckhard et al., 2015
MMP-25	Role remains unclear	Eckhard et al., 2015

## List of Abbreviations

BSP2: bone sialoprotein
CTGF: connective-tissue growth factor
DEJ: dentineoenamel junction
DMP1: dentine-matrix protein 1
DPP: dentine phosphoprotein
DSP: dentinesialoprotein
DSPP: dentine sialophosphoprotein
EDTA: ethylenediaminetetraacetic acid
FGF2: fibroblast growth factor 2
MEPE: matrix extracellular phosphoglycoprotein
MMP: matrix metalloproteinase
OPN: osteopontin
PDGF-AB: platelet-derived growth factor AB
PlGF: placenta growth factor
SIBLING: small integrin-binding ligand, N-linked glycoprotein
TGF-β1: transforming growth factor-β1
TIMP: tissue inhibitors of metalloproteinases
VEGF: vascular endothelial growth factor

## References

[R1] Abbass MMS, El-Rashidy AA, Sadek KM, Moshy SE, Radwan IA, Rady D, Dörfer CE, Fawzy El-Sayed KM (2020) Hydrogels and dentin-pulp complex regeneration: from the benchtop to clinical translation. Polymers (Basel) 12: 2935. DOI: 10.3390/polym12122935. PMC7763835 33316886

[R2] Abou Neel EA, Aljabo A, Strange A, Ibrahim S, Coathup M, Young AM, Bozec L, Mudera V (2016) Demineralization–remineralization dynamics in teeth and bone. Int J Nanomedicine 11: 4743–4763. 27695330 10.2147/IJN.S107624 PMC5034904

[R3] Almushayt A, Narayanan K, Zaki AE, George A (2006) Dentin matrix protein 1 induces cytodifferentiation of dental pulp stem cells into odontoblasts. Gene Ther 13: 611–620. 16319946 10.1038/sj.gt.3302687

[R4] Bègue-Kirn C, Smith AJ, Ruch JV, Wozney JM, Purchio A, Hartmann D, Lesot H (2004) Effects of dentin proteins, transforming growth factor beta 1 (TGF beta 1) and bone morphogenetic protein 2 (BMP2) on the differentiation of odontoblast *in vitro*. Int J Dev Biol 36: 491–503. 1295560

[R5] Bourd-Boittin K, Fridman R, Fanchon S, Septier D, Goldberg M, Menashi S (2005) Matrix metalloproteinase inhibition impairs the processing, formation and mineralization of dental tissues during mouse molar development. Exp Cell Res 304: 493–505. 15748894 10.1016/j.yexcr.2004.11.024

[R6] Breschi L, Maravic T, Cunha SR, Comba A, Cadenaro M, Tjäderhane L, Pashley DH, Tay FR, Mazzoni A (2018) Dentin bonding systems: from dentin collagen structure to bond preservation and clinical applications. Dent Mater 34: 78–96. 29179971 10.1016/j.dental.2017.11.005

[R7] Chang YC, Chang MC, Chen YJ, Liou JU, Chang HH, Huang WL, Liao WC, Chan CP, Jeng PY, Jeng JH (2017) Basic fibroblast growth factor regulates gene and protein expression related to proliferation, differentiation, and matrix production of human dental pulp cells. J Endod 43: 936–942. 28416318 10.1016/j.joen.2017.01.024

[R8] Charadram N, Farahani RM, Harty D, Rathsam C, Swain MV, Hunter N (2012) Regulation of reactionary dentin formation by odontoblasts in response to polymicrobial invasion of dentin matrix. Bone 50: 265–275. 22079283 10.1016/j.bone.2011.10.031 PMC3246533

[R9] Charadram N, Austin C, Trimby P, Simonian M, Swain MV, Hunter N (2013) Structural analysis of reactionary dentin formed in response to polymicrobial invasion. J Struct Biol 181: 207–222. 23261402 10.1016/j.jsb.2012.12.005 PMC3578079

[R10] Chaussain C, Eapen AS, Huet E, Floris C, Ravindran S, Hao J, Menashi S, George A (2009) MMP2-cleavage of DMP1 generates a bioactive peptide promoting differentiation of dental pulp stem/progenitor cells. Eur Cell Mater 18: 84–95. 19908197 10.22203/ecm.v018a08 PMC3092783

[R11] Chaussain-Miller C, Fioretti F, Goldberg M, Menashi S (2006) The role of matrix metalloproteinases (MMPs) in human caries. J Dent Res 85: 22–32. 16373676 10.1177/154405910608500104

[R12] Eapen A, George A (2015) Dentin phosphophoryn in the matrix activates AKT and mTOR signaling pathway to promote preodontoblast survival and differentiation. Front Physiol 6: 221. DOI: 10.3389/fphys.2015.00221 26300786 PMC4528161

[R13] Eba H, Murasawa Y, Iohara K, Isogai Z, Nakamura H, Nakamura H, Nakashima M (2012) The anti-inflammatory effects of matrix metalloproteinase-3 on irreversible pulpitis of mature erupted teeth. PLoS One 7: e52523. DOI: 10.1371/journal.pone.0052523. 23285075 PMC3527558

[R14] Eckhard U, Marino G, Abbey SR, Tharmarajah G, Matthew I, Overall CM (2015) The human dental pulp proteome and N-terminome: levering the unexplored potential of semitryptic peptides enriched by TAILS to identify missing proteins in the human proteome project in underexplored tissues. J Proteome Res 14: 3568–3582. 26258467 10.1021/acs.jproteome.5b00579

[R15] Fedarko NS, Jain A, Karadag A, Fisher LW (2004) Three small integrin binding ligand N-linked glycoproteins (SIBLINGs) bind and activate specific matrix metalloproteinases. FASEB J 18: 734–736. 14766790 10.1096/fj.03-0966fje

[R16] Galler KM, D’Souza RN, Federlin M, Cavender AC, Hartgerink JD, Hecker S, Schmalz G (2011) Dentin conditioning codetermines cell fate in regenerative endodontics. J Endod 37: 1536–1541. 22000458 10.1016/j.joen.2011.08.027

[R17] García-Bernal D, López-García S, Sanz JL, Guerrero-Gironés J, García-Navarro EM, Moraleda JM, Forner L, Rodríguez-Lozano FJ (2020) Melatonin treatment alters biological and immunomodulatory properties of human dental pulp mesenchymal stem cells *via* augmented TGFβ secretion. J Endod 47: 424–435. 33359532 10.1016/j.joen.2020.12.008

[R18] Gendron R, Grenier D, Sorsa T, Mayrand D (1999) Inhibition of the activities of matrix metalloproteinases 2, 8, and 9 by chlorhexidine. Clin Diagn Lab Immunol 6: 437–439. 10225852 10.1128/cdli.6.3.437-439.1999 PMC103739

[R19] Goldberg M, Septier D, Bourd K, Hall R, George A, Goldberg H, Menashi S (2003) Immunohistochemical localization of MMP-2, MMP-9, TIMP-1, and TIMP-2 in the forming rat incisor. Connect Tissue Res 44: 143–153. 14504034 10.1080/03008200390223927

[R20] Goldberg M, Kulkarni AB, Young M, Boskey A (2011) Dentin: structure, composition and mineralization. Front Biosci (Elite Ed) 3: 711–735. 21196346 10.2741/e281 PMC3360947

[R21] Goldberg M, Njeh A, Uzunoglu E (2015) Is pulp inflammation a prerequisite for pulp healing and regeneration? Mediators Inflamm 2015: 347649. DOI: 10.1155/2015/347649. 26538825 PMC4619968

[R22] Gusman H, Santana RB, Zehnder M (2002) Matrix metalloproteinase levels and gelatinolytic activity in clinically healthy and inflamed human dental pulps. Eur J Oral Sci 110: 353–357. 12664465 10.1034/j.1600-0722.2002.21347.x

[R23] Hall R, Septier D, Embery G, Goldberg M (1999) Stromelysin-1 (MMP-3) in forming enamel and predentine in rat incisor – coordinated distribution with proteoglycans suggests a functional role. Histochem J 31: 761–770. 10661319 10.1023/a:1003945902473

[R24] Hanemaaijer R, van Lent N, Sorsa T, Salo T, YrjŐ, Konttinen T, Lindeman J (2001) Inhibition of matrix metalloproteinases (MMPs) by tetracyclines. In: Tetracyclines in Biology, Chemistry and Medicine. Editors: Nelson M, Hillen W, Greenwald RA. Basel, Birkhäuser. pp: 267–281.

[R25] Heikinheimo K, Salo T (1995) Expression of basement membrane type IV collagen and type IV collagenases (MMP-2 and MMP-9) in human fetal teeth. J Dent Res 74: 1226–1234. 7790601 10.1177/00220345950740051301

[R26] Hong LTA, Kim YM, Park HH, Hwang DH, Cui Y, Lee EM, Yahn S, Lee JK, Song SC, Kim BG (2017) An injectable hydrogel enhances tissue repair after spinal cord injury by promoting extracellular matrix remodeling. Nat Commun 8: 533. DOI: 10.1038/s41467-017-00583-8. 28912446 PMC5599609

[R27] Huang GT (2011) Dental pulp and dentin tissue engineering and regeneration: advancement and challenge. Front Biosci (Elite Ed) 3: 788–800. 21196351 10.2741/e286 PMC3289134

[R28] Huang CC, Ravindran S, Kang M, Cooper LF, George A (2020) Engineering a self-assembling leucine zipper hydrogel system with function-specific motifs for tissue regeneration. ACS Biomater Sci Eng 6: 2913–2928. 33463282 10.1021/acsbiomaterials.0c00026 PMC8080846

[R29] Inuyama Y, Kitamura C, Nishihara T, Morotomi T, Nagayoshi M, Tabata Y, Matsuo K, Chen KK, Terashita M (2010) Effects of hyaluronic acid sponge as a scaffold on odontoblastic cell line and amputated dental pulp. J Biomed Mater Res B Appl Biomater 92: 120–128. 19802830 10.1002/jbm.b.31497

[R30] Ishimatsu H, Kitamura C, Morotomi T, Tabata Y, Nishihara T, Chen KK, Terashita M (2009) Formation of dentinal bridge on surface of regenerated dental pulp in dentin defects by controlled release of fibroblast growth factor–2 from gelatin hydrogels. J Endod 35: 858–865. 19482186 10.1016/j.joen.2009.03.049

[R31] Jontell M, Linde A (1983) Non-collagenous proteins of predentine from dentinogenically active bovine teeth. Biochem J 214: 769–776. 6626156 10.1042/bj2140769 PMC1152314

[R32] Koh JW, Shin YJ, Oh JY, Kim MK, Ko JH, Hwang JM, Wee WR, Lee JH (2007) The expression of TIMPs in cryo-preserved and freeze-dried amniotic membrane. Curr Eye Res 32: 611–616. 17852184 10.1080/02713680701459441

[R33] Krafts KP (2010) Tissue repair: the hidden drama. Organogenesis 6 225–233. 21220961 10.4161/org.6.4.12555 PMC3055648

[R34] Liu L, Leng S, Tang L, Lu Q, Xu W, Tan X, Huang D, Zhang L (2021) EDTA promotes the mineralization of dental pulp *in vitro* and *in vivo*. J Endod 47: 458–465. 33352150 10.1016/j.joen.2020.12.003

[R35] Löffek S, Schilling O, Franzke CW (2011) Series “matrix metalloproteinases in lung health and disease”: biological role of matrix metalloproteinases: a critical balance. Eur Respir J 38: 191–208. 21177845 10.1183/09031936.00146510

[R36] Loreto C, Galanti C, Musumeci G, Rusu MC, Leonardi R (2014) Immunohistochemical analysis of matrix metalloproteinase-13 in human caries dentin. Eur J Histochem 58: 2318. DOI: 10.4081/ejh.2014.2318. 24704999 PMC3980212

[R37] Lutolf MP, Lauer-Fields JL, Schmoekel HG, Metters AT, Weber FE, Fields GB, Hubbell JA (2003) Synthetic matrix metalloproteinase-sensitive hydrogels for the conduction of tissue regeneration: engineering cell-invasion characteristics. Proc Natl Acad Sci U S A 100: 5413–5418. 12686696 10.1073/pnas.0737381100 PMC154359

[R38] MacDougall M, Slavkin HC, Zeichner-David M (1992) Characteristics of phosphorylated and non-phosphorylated dentine phosphoprotein. Biochem J 287 (Pt 2): 651–655. 1445225 10.1042/bj2870651 PMC1133215

[R39] Mazzoni A, Mannello F, Tay FR, Tonti GA, Papa S, Mazzotti G, Di Lenarda R, Pashley DH, Breschi L (2007) Zymographic analysis and characterization of MMP-2 and -9 forms in human sound dentin. J Dent Res 86: 436–440. 17452564 10.1177/154405910708600509

[R40] Mazzoni A, Maravić T, Tezvergil-Mutluay A, Tjäderhane L, Scaffa PMC, Seseogullari-Dirihan R, Bavelloni A, Gobbi P, Pashley DH, Tay FR, Breschi L (2018) Biochemical and immunohistochemical identification of MMP-7 in human dentin. J Dent 79: 90–95. 30367893 10.1016/j.jdent.2018.10.008

[R41] Mente J, Petrovic J, Gehrig H, Rampf S, Michel A, Schürz A, Pfefferle T, Saure D, Erber R (2016) A prospective clinical pilot study on the level of matrix metalloproteinase-9 in dental pulpal blood as a marker for the state of inflammation in the pulp tissue. J Endod 42: 190–197. 26725178 10.1016/j.joen.2015.10.020

[R42] Miyazaki K, Uchiyama K, Imokawa Y, Yoshizato K (1996) Cloning and characterization of cDNAs for matrix metalloproteinases of regenerating newt limbs. Proc Natl Acad Sci U S A 93: 6819–6824. 8692902 10.1073/pnas.93.13.6819 PMC39111

[R43] Muromachi K, Kamio N, Narita T, Annen-Kamio M, Sugiya H, Matsushima K (2012) MMP-3 provokes CTGF/CCN2 production independently of protease activity and dependently on dynamin-related endocytosis, which contributes to human dental pulp cell migration. J Cell Biochem 113: 1348–1358. 22134873 10.1002/jcb.24007

[R44] Murphy CM, O’Brien FJ, Little DG, Schindeler A (2013) Cell-scaffold interactions in the bone tissue engineering triad. Eur Cell Mater 26: 120–132. 24052425 10.22203/ecm.v026a09

[R45] Nakashima M (1994) Induction of dentin formation on canine amputated pulp by recombinant human bone morphogenetic proteins (BMP)-2 and -4. J Dent Res 73: 1515–1522. 7929986 10.1177/00220345940730090601

[R46] Ogbureke KU, Fisher LW (2004) Expression of SIBLINGs and their partner MMPs in salivary glands. J Dent Res 83: 664–670. 15329369 10.1177/154405910408300902

[R47] Okamoto M, Takahashi Y, Komichi S, Cooper PR, Hayashi M (2018) Dentinogenic effects of extracted dentin matrix components digested with matrix metalloproteinases. Sci Rep 8: 10690. DOI: 10.1038/s41598-018-29112-3. 30013085 PMC6048071

[R48] Ortega N, Behonick D, Stickens D, Werb Z (2003) How proteases regulate bone morphogenesis. Ann N Y Acad Sci 995: 109–116. 12814943 10.1111/j.1749-6632.2003.tb03214.x

[R49] Pandya M, Diekwisch TGH (2019) Enamel biomimetics-fiction or future of dentistry. Int J Oral Sci 11: 8. DOI: 10.1038/s41368-018-0038-6. 30610185 PMC6320371

[R50] Panseri S, Montesi M, Dozio SM, Savini E, Tampieri A, Sandri M (2016) Biomimetic scaffold with aligned microporosity designed for dentin regeneration. Front Bioeng Biotechnol 4: 48. DOI: 10.3389/fbioe.2016.00048. 27376060 PMC4896952

[R51] Pashley D, Okabe A, Parham P (1985) The relationship between dentin microhardness and tubule density. Endod Dent Traumatol 1: 176–179. 3865764 10.1111/j.1600-9657.1985.tb00653.x

[R52] Prescott RS, Alsanea R, Fayad MI, Johnson BR, Wenckus CS, Hao J, John AS, George A (2008) *In vivo* generation of dental pulp-like tissue using human pulpal stem cells, a collagen scaffold and dentin matrix protein 1 following subcutaneous transplantation in mice. J Endod 34: 421–426. 18358888 10.1016/j.joen.2008.02.005 PMC2408448

[R53] Ratnikov BI, Cieplak P, Gramatikoff K, Pierce J, Eroshkin A, Igarashi Y, Kazanov M, Sun Q, Godzik A, Osterman A, Stec B, Strongin A, Smith JW (2014) Basis for substrate recognition and distinction by matrix metalloproteinases. Proc Natl Acad Sci U S A 111: E4148–E4155. 25246591 10.1073/pnas.1406134111 PMC4210027

[R54] Roberts-Clark DJ, Smith AJ (2000) Angiogenic growth factors in human dentine matrix. Arch Oral Biol 45: 1013–1016. 11000388 10.1016/s0003-9969(00)00075-3

[R55] Sadaghiani L, Gleeson HB, Youde S, Waddington RJ, Lynch CD, Sloan AJ (2016) Growth factor liberation and DPSC response following dentine conditioning. J Dent Res 95: 1298–1307. 27307049 10.1177/0022034516653568

[R56] Senawongse P, Otsuki M, Tagami J, Mjör I (2006) Age-related changes in hardness and modulus of elasticity of dentine. Arch Oral Biol 51: 457–463. 16426564 10.1016/j.archoralbio.2005.11.006

[R57] Sharma R, Kumar V, Logani A, Chawla A, Mir RA, Sharma S, Kalaivani M (2020) Association between concentration of active MMP-9 in pulpal blood and pulpotomy outcome in permanent mature teeth with irreversible pulpitis – a preliminary study. Int Endod J 54: 479–489. 33128238 10.1111/iej.13437

[R58] Sternlicht MD, Werb Z (2001) How matrix metalloproteinases regulate cell behavior. Annu Rev Cell Dev Biol 17: 463–516. 11687497 10.1146/annurev.cellbio.17.1.463 PMC2792593

[R59] Sulkala M, Larmas M, Sorsa T, Salo T, Tjäderhane L (2002) The localization of matrix metalloproteinase-20 (MMP-20, enamelysin) in mature human teeth. J Dent Res 81: 603–607. 12202640 10.1177/154405910208100905

[R60] Sulkala M, Tervahartiala T, Sorsa T, Larmas M, Salo T, Tjäderhane L (2007) Matrix metalloproteinase-8 (MMP-8) is the major collagenase in human dentin. Arch Oral Biol 52: 121–127. 17045563 10.1016/j.archoralbio.2006.08.009

[R61] Suresh N, Arul B, Kowsky D, Natanasabapathy V (2018) Successful regenerative endodontic procedure of a nonvital immature permanent central incisor using amniotic membrane as a novel scaffold. Dent J (Basel) 6: 36. DOI: 10.3390/dj6030036. PMC6162468 30072584

[R62] Tannure PN, Küchler EC, Falagan-Lotsch P, Amorim LM, Raggio Luiz R, Costa MC, Vieira AR, Granjeiro JM (2012) MMP13 polymorphism decreases risk for dental caries. Caries Res 46: 401–407. 22710194 10.1159/000339379

[R63] Tezvergil-Mutluay A, Mutluay M, Seseogullari-Dirihan R, Agee KA, Key WO, Scheffel DL, Breschi L, Mazzoni A, Tjäderhane L, Nishitani Y, Tay FR, Pashley DH (2013) of phosphoric acid on the degradation of human dentin matrix. J Dent Res 92: 87–91. 23103634 10.1177/0022034512466264 PMC3521452

[R64] Tjäderhane L, Buzalaf MA, Carrilho M, Chaussain C (2015) Matrix metalloproteinases and other matrix proteinases in relation to cariology: the era of ‘dentin degradomics’. Caries Res 49: 193–208. 25661522 10.1159/000363582

[R65] Turturro MV, Christenson MC, Larson JC, Young DA, Brey EM, Papavasiliou G (2013) MMP-sensitive PEG diacrylate hydrogels with spatial variations in matrix properties stimulate directional vascular sprout formation. PLoS One 8: e58897. DOI: 10.1371/journal.pone.0058897. 23554954 PMC3595229

[R66] Vagropoulou G, Trentsiou M, Georgopoulou A, Papachristou E, Prymak O, Kritis A, Epple M, Chatzinikolaidou M, Bakopoulou A, Koidis P (2021) Hybrid chitosan/gelatin/nanohydroxyapatite scaffolds promote odontogenic differentiation of dental pulp stem cells and *in vitro* biomineralization. Dent Mater 37: e23–e36. 33208264 10.1016/j.dental.2020.09.021

[R67] Van Wart HE, Birkedal-Hansen H (1990) The cysteine switch: a principle of regulation of metalloproteinase activity with potential applicability to the entire matrix metalloproteinase gene family. Proc Natl Acad Sci U S A 87: 5578–5582. 2164689 10.1073/pnas.87.14.5578 PMC54368

[R68] VanHook A (2008) Novel function for a matrix metalloprotease. Sci Signal 1: ec103–ec103.

[R69] Vinarsky V, Atkinson DL, Stevenson TJ, Keating MT, Odelberg SJ (2005) Normal newt limb regeneration requires matrix metalloproteinase function. Dev Biol 279: 86–98. 15708560 10.1016/j.ydbio.2004.12.003

[R70] Wang RZ, Weiner S (1997) Strain-structure relations in human teeth using Moiré fringes. J Biomech 31: 135–141. 10.1016/s0021-9290(97)00131-0 9593206

[R71] Wilhelm SM, Shao ZH, Housley TJ, Seperack PK, Baumann AP, Gunja-Smith Z, Woessner JF Jr (1993) Matrix metalloproteinase-3 (stromelysin-1). Identification as the cartilage acid metalloprotease and effect of pH on catalytic properties and calcium affinity. J Biol Chem 268: 21906–21913. 8408046

[R72] Xu F, Qiao L, Zhao Y, Chen W, Hong S, Pan J, Jiang B (2019) The potential application of concentrated growth factor in pulp regeneration: an *in vitro* and *in vivo* study. Stem Cell Res Ther 10: 134. DOI: 10.1186/s13287-019-1247-4. 31109358 PMC6528367

[R73] Xu H, Snider TN, Wimer HF, Yamada SS, Yang T, Holmbeck K, Foster BL (2016) Multiple essential MT1-MMP functions in tooth root formation, dentinogenesis, and tooth eruption. Matrix Biol 52-54: 266–283. 26780723 10.1016/j.matbio.2016.01.002 PMC4875876

[R74] Yang JW, Zhang YF, Sun ZY, Song GT, Chen Z (2015) Dental pulp tissue engineering with bFGF-incorporated silk fibroin scaffolds. J Biomater Appl 30: 221–229. 25791684 10.1177/0885328215577296

[R75] Yang KC, Wang CH, Chang HH, Chan WP, Chi CH, Kuo TF (2012) Fibrin glue mixed with platelet-rich fibrin as a scaffold seeded with dental bud cells for tooth regeneration. J Tissue Eng Regen Med 6: 777–785. 22034398 10.1002/term.483

[R76] Ye Q, van Amerongen MJ, Sandham JA, Bank RA, van Luyn MJ, Harmsen MC (2011) Site-specific tissue inhibitor of metalloproteinase-1 governs the matrix metalloproteinases-dependent degradation of crosslinked collagen scaffolds and is correlated with interleukin-10. J Tissue Eng Regen Med 5: 264–274. 20661871 10.1002/term.311

[R77] Yuan G, Chen L, Feng J, Yang G, Ni Q, Xu X, Wan C, Lindsey M, Donly KJ, MacDougall M, Chen Z, Chen S (2017) Dentin sialoprotein is a novel substrate of matrix metalloproteinase 9 *in vitro* and *in vivo*. Sci Rep 7: 42449. DOI: 10.1038/srep42449. 28195206 PMC5307955

[R78] Zehnder M, Wegehaupt FJ, Attin T (2011) A first study on the usefulness of matrix metalloproteinase 9 from dentinal fluid to indicate pulp inflammation. J Endod 37: 17–20. 21146069 10.1016/j.joen.2010.10.003

[R79] Zhang M, Jiang F, Zhang X, Wang S, Jin Y, Zhang W, Jiang X (2017) The effects of platelet-derived growth factor-BB on human dental pulp stem cells mediated dentin-pulp complex regeneration. Stem Cells Transl Med 6: 2126–2134. 29064632 10.1002/sctm.17-0033 PMC5702518

[R80] Zhao S, Sloan AJ, Murray PE, Lumley PJ, Smith AJ (2000) Ultrastructural localisation of TGF-beta exposure in dentine by chemical treatment. Histochem J 32: 489–494. 11095074 10.1023/a:1004100518245

[R81] Zheng L, Amano K, Iohara K, Ito M, Imabayashi K, Into T, Matsushita K, Nakamura H, Nakashima M (2009) Matrix metalloproteinase-3 accelerates wound healing following dental pulp injury. Am J Pathol 175: 1905–1914. 19834065 10.2353/ajpath.2009.080705 PMC2774055

